# Supplementary material for: Inductively shunted transmons exhibit noise insensitive plasmon states and a fluxon decay exceeding 3 hours
Source: Nat Commun. 2023 Jul 5;14:3968. doi: 10.1038/s41467-023-39656-2 (PMC10323121; doi:10.1038/s41467-023-39656-2)
Supplement: Supplementary file 1 — Supplementary Information [file 41467_2023_39656_MOESM1_ESM.pdf]

Supplementary Information for
Inductively shunted transmons exhibit noise insensitive plasmon states and a fluxon decay exceeding 3 hours

F. Hassan,† M. Peruzzo, L. N. Kapoor, A. Trioni, M. Zemlicka, and J. M. Fink‡
Institute of Science and Technology Austria, Am Campus 1, 3400 Klosterneuburg, Austria
(Dated: June 21, 2023)

SUPPLEMENTARY NOTE 1: PERTURBATION THEORY

We use perturbation theory to investigate the degree of protection characterized by the E_J/E_L ratio. We split the Hamiltonian in equation (1) of the main text into the transmon part and an inductive part that we treat as a perturbation

$$\begin{aligned}\hat{H} &= \hat{H}_{\text{trans}} + \hat{H}_{\text{per}},\\ \hat{H}_{\text{trans}} &= 4E_C\hat{n}^2 - E_J\cos\hat{\phi},\\ \hat{H}_{\text{per}} &= \frac{1}{2}E_L(\hat{\phi}+\varphi_{\text{ext}})^2.\end{aligned} \tag{1}$$

Then, by following the formalism of perturbation theory [1], we can calculate the corrections to transmon eigenenergies up to second order

$$E_m = E_m^{(0)} + E_m^{(1)} + E_m^{(2)}, \tag{2}$$

where $E_m^{(0)}$ being the eigenenergies of the transmon derived from Mathieu functions [2] and $E_m^{(1)}$ and $E_m^{(2)}$ representing the first and second order energy correction respectively. These values can be calculated using

$$\begin{aligned}E_m^{(1)} &= \langle m|\hat{H}_{\text{per}}|m\rangle\,,\\ E_m^{(2)} &= \sum_{m\neq n}\frac{\left|\langle n|\hat{H}_{\text{per}}|m\rangle\right|^2}{E_m^{(0)}-E_n^{(0)}}.\end{aligned} \tag{3}$$

Now, to find an expression for $E_m^{(1)}$ and $E_m^{(2)}$, we employ the second quantization formalism of the flux operator

$$\hat{\phi} = \left(\frac{2E_C}{E_J}\right)^{\frac{1}{4}}\left(\hat{a}^\dagger + \hat{a}\right), \tag{4}$$

$$\hat{a}^\dagger|n\rangle = \sqrt{n+1}|n+1\rangle, \tag{5}$$

$$\hat{a}|n\rangle = \sqrt{n}|n-1\rangle, \tag{6}$$

with $\hat{a}^\dagger$ and $\hat{a}$ being the raising and lowering operators for the transmon and n is the transmon qubit state number. Substituting equation (4) in the first order energy correction term shown in equation (3), we obtain

$$E_n^{(1)} = \frac{1}{2\hbar}\left(2n+1\right)E_L\left(\frac{2E_C}{E_J}\right)^{\frac{1}{2}} + \frac{1}{2\hbar}E_L\varphi_{\text{ext}}^2. \tag{7}$$

In the first order energy correction shown in equation (7), the external flux appears with a prefactor that is not state number dependent. Consequently, the calculated transition energies using only the first order correction will not

---

† farid.hassani@ist.ac.at

‡ jfink@ist.ac.at

reflect the external flux effect on the transition. Therefore, we extend the calculations to second order terms. The expression for ground and excited second order energy corrections are

$$E_0^{(2)} = \frac{1}{4\hbar} E_L^2 \left[\frac{2\left(\frac{2E_C}{E_J}\right)}{E_0^{(0)} - E_2^{(0)}} + \frac{4\left(\frac{2E_C}{E_J}\right)^{\frac{1}{2}} \varphi_{\text{ext}}^2}{E_0^{(0)} - E_1^{(0)}} \right],$$

$$E_1^{(2)} = \frac{1}{4\hbar} E_L^2 \left(\frac{2E_C}{E_J}\right) \left[\frac{6}{E_1^{(0)} - E_3^{(0)}} \right] +$$
$$\frac{1}{4\hbar} E_L^2 \left(\frac{2E_C}{E_J}\right)^{\frac{1}{2}} \left[\frac{8\varphi_{\text{ext}}^2}{E_1^{(0)} - E_2^{(0)}} + \frac{4\varphi_{\text{ext}}^2}{E_1^{(0)} - E_0^{(0)}} \right], \tag{8}$$

while the general expression for $n \gg 2$ is

$$E_{n\geq 2}^{(2)} = \frac{E_L^2}{\hbar} \left(\frac{E_C}{2E_J}\right) \left[\frac{(n+1)(n+2)}{E_n^{(0)} - E_{n+2}^{(0)}} + \frac{(n)(n-1)}{E_n^{(0)} - E_{n-2}^{(0)}} \right] +$$
$$\frac{E_L^2}{\hbar} \left(\frac{2E_C}{E_J}\right)^{\frac{1}{2}} \varphi_{\text{ext}}^2 \left[\frac{(n+1)}{E_n^{(0)} - E_{n+1}^{(0)}} + \frac{n}{E_n^{(0)} - E_{n-1}^{(0)}} \right]. \tag{9}$$

The second order energy correction terms are also dependent to φ_{ext} but with a prefactor determined by the transmon state number. In Supplementary Figure 1 the result of a numerical calculation of a typical IST qubit's first transition frequency with $E_J/h = 35, E_C/h = 0.15, E_L/h = 2$ (all in GHz) is plotted against a prediction from perturbation theory. The analytic solution has inaccuracies both in frequency and dispersion but for small E_L the results converge to the exact numerical solution as shown in the inset of Supplementary Figure 1).

Finally, to arrive at equation (2) in the main text, we calculate the derivative of the first transition

$$\frac{\partial \omega_{p01}}{\partial \varphi_{\text{ext}}} =$$
$$4\frac{E_L^2}{\hbar} \varphi_{\text{ext}} \left(\frac{2E_c}{E_J}\right)^{\frac{1}{2}} \left[\frac{1}{E_1^{(0)} - E_2^{(0)}} + \frac{1}{E_1^{(0)} - E_0^{(0)}} \right]. \tag{10}$$

Now, using the approximation of $E_1^{(0)} - E_2^{(0)} = -\sqrt{8E_J E_C} + 2E_C$ and $E_1^{(0)} - E_0^{(0)} = \sqrt{8E_J E_C} - E_C$ in the limit of high E_J/E_C, we can further simplify the expression in equation (10) to

$$\frac{\partial \omega_{p01}}{\partial \varphi_{\text{ext}}} = 4\frac{E_L^2}{\hbar} \varphi_{\text{ext}} \left(\frac{2E_C}{E_J}\right)^{\frac{1}{2}} \times$$
$$\left[\frac{-E_C}{\left(\sqrt{8E_J E_C} - 2E_C\right)\left(\sqrt{8E_J E_C} - E_C\right)} \right], \tag{11}$$

which, by ignoring the E_C terms in the denominator in comparison with plasmon frequency $\sqrt{8E_J E_C}$, can be further simplified to

$$\frac{\partial \omega_{p01}}{\partial \varphi_{\text{ext}}} \approx -\frac{\sqrt{8E_J E_C}}{\hbar (2E_J/E_L)^2} \varphi_{\text{ext}}. \tag{12}$$

The second order derivative can be calculated from equation (12) as

$$\frac{\partial^2 \omega_{p01}}{\partial {\varphi_{\text{ext}}}^2} \approx -\frac{\sqrt{8E_J E_C}}{\hbar (2E_J/E_L)^2}. \tag{13}$$

It is important to mention that this theoretical description actually models a periodic parabolic potential as the perturbation and only represents our system (the IST qubit) in a local sense, i.e. within the first flux quantum and for $\phi \in (-\pi, \pi)$. Without taking this into consideration the wavefunctions obtained from perturbation theory will not be periodic and therefore contradict the periodic transmon wavefunctions. Beyond the first flux quantum perturbation theory fails to predict the IST qubit properties simply because its potential is different than that of the IST qubit.

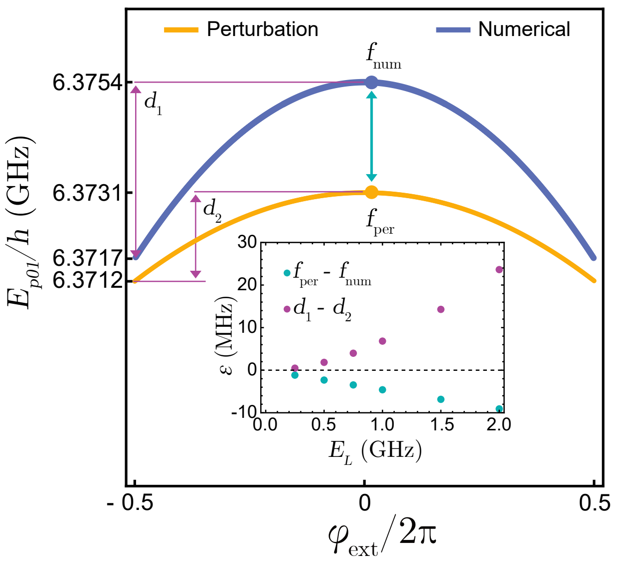

Supplementary Figure. 1. **Plasmon transition energy.** Typical ground to excited state transition frequency of the IST qubit with $E_J/h = 35$, $E_C/h = 0.15$ and $E_L/h = 0.5\,\text{GHz}$, calculated numerically (blue) and predicted using perturbation theory (yellow). The analytic solution shows a deviation ϵ in predicting the frequency (green arrow) and dispersion (difference of purple arrows). The inset shows how these errors scale with the perturbation strength E_L. As the inductive energy decreases the error in both frequency and dispersion converge to the numerical results.

SUPPLEMENTARY NOTE 2: DEVICE FABRICATION

The fabrication of the IST qubit starts with cleaning a 10×10 mm^2 high resistivity silicon chip using an O_2 plasma asher followed by a buffered hydrofluoric acid dip, sonication in acetone for 10 minutes at a temperature of 50°C and a final rinse with isopropanol (IPA). To form the alignment markers and identifiers, the chip was covered with AR-P 6200 (CSAR 62) resist and patterned to dry etch with an Oxford ICP machine. After ICP dry etching, the chip is solvent cleaned with N-Methylpyrrolidone (NMP) followed by an acetone and IPA rinse.

In the next layer, the CSAR 62 was also used to lift-off the first layer of aluminium with a thickness of 100 nm forming the cross-wire (shown in Supplementary Figure 2a) which provides access to the inner pad of the geometric superinductor in the subsequent layers. To protect the cross-wire and to shape the air-bridges as smooth arcs as shown in Fig. 4, the CSAR 62 was patterned and reflowed at 180°C (Supplementary Figure 2b). Then 150 nm of aluminium was evaporated while the chip was tilted at 10 degrees and the sample holder was in rotation (Supplementary Figure 2c). The tilt and rotation helps to cover the resist in the previous step uniformly. Subsequently, the aluminium layer was dry etched using a calibrated mixture of BCl_3 - Cl_2 gases to form the geometric superinductor and capacitive antenna pads (Supplementary Figure 2d and e). Then the protective resist was cleaned in NMP solvent (Supplementary Figure 2f) in preparation for the standard Josephson junction fabrication.

Next, the sample was covered with a double layer of MMA/PMMA resist to pattern the Dolan bridge [3] for Josephson-junction shadow evaporation. Before junction evaporation starts, we use an in-situ gentle argon ion milling process to clean the surface of the silicon of possible resist residues. The process uses a 250 V acceleration voltage and a current of 10 mA with an argon flow of 4.5 sccm. Then the Josephson junction was fabricated by first evaporating 60 nm aluminium as the base electrode of the junction followed by a calibrated static oxidation with oxygen at a pressure of 5 mbar and 5 minutes using pure O_2 for a Josephson energy of 35 GHz (with an area of 250×250 nm^2), and finally the top electrode of the junction was evaporated with a thickness of 120 nm.

The final layer of the device connects all the previous layers with a suitable conductive patch. To remove the aluminium oxide we use in-situ argon ion milling with more aggressive parameters (400 V, 21 mA and 4.5 sccm argon flow) for 5 minutes. Since this layer has to cover all the previous layers, a 300 nm thick aluminium film was used. A double layer PMMA resist with a thickness of approximately 1 μm was used to assist the final lift-off process. Finally, the chip was covered with a S1805 photo resist and a UV tape to be diced into three 10×2.5 mm^2 pieces each hosting one qubit device.

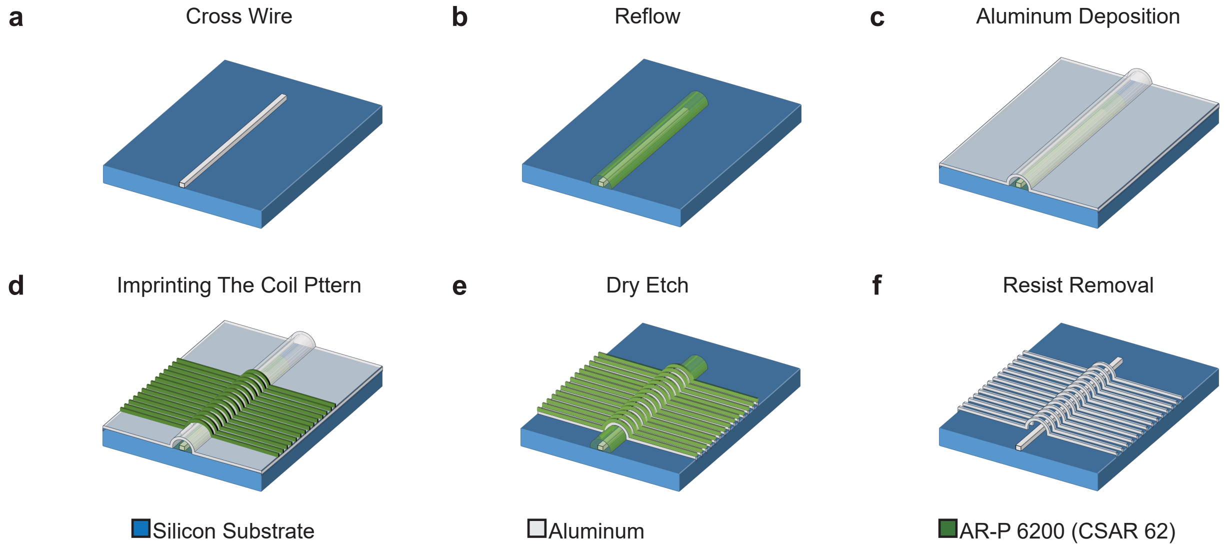

Supplementary Figure. 2. **Fabrication steps for the etched geometric superinductor.** **a**, Shows the cross-wire layer used to access the inner electrode of the geometric superinductor. **b**, Shows the CSAR 62 patterned and reflowed at 180°C. The resist will protect the cross wire from the etching and shapes the bridges. The resist was spun at 6000 rpm for one minute to reach a height of about 250 to 300 nm. The height of the bridge is an important factor for their stability. **c**, Aluminium deposition with a thickness of 150 nm. The substrates was tilted at 10 degrees and rotating during the evaporation to ensure a uniform thickness covering the arc shaped resist. **d**, Exposure and development of the inverse coil pattern with the protective resist CSAR 62, which is spun at 1500 rpm to ensure that it covers the arcs and withstands the etching step. **e**, Dry etching the aluminium with an inductively coupled plasma (ICP) machine using a mixture of BCl_3 - Cl_2 gases. **f**, Removal of the protecting resist with NMP solvent.

SUPPLEMENTARY NOTE 3: MEASUREMENT SETUP

In Supplementary Supplementary Figure 3 we show the experimental setup with the DC and microwave components inside the cryostat used to perform all presented experiments.

SUPPLEMENTARY NOTE 4: FITTING PROCEDURE

To fit the spectroscopy data shown in Fig. 5 of the main text, we numerically solve (using the scQubits library) a fluxonium in the IST regime coupled to a resonator and extract the eigenenergies as shown in Supplementary Figure 4a. At zero flux bias, the first transition is labelled correctly by $|p0\rangle \rightarrow |p1\rangle$ (position a in Supplementary Figure 4a), however, since the flux transition is not allowed in the IST qubit and the phase particle is trapped within one well, the experimentally measured first transition at position b refers to $|p0\rangle \rightarrow |p2\rangle$ while the same transition at position c refers to $|p1\rangle \rightarrow |p3\rangle$. The constant change in state numbers poses a challenge to fit the experimental data.

In Supplementary Figure 4a, one may notice that the eigenenergies for any state is a quadratic function of external flux. The intuition for this observation is provided in [4], where applying a transformation to the Hamiltonian of equation (1) of the main text into the Bloch wave basis and in the limit of high E_J/E_C ratio, block diagonalizes the Hamiltonian into separate effective Hamiltonians expressed as

$$H^{(s)} = \frac{E_L}{2}\left(i\frac{d}{dp} + \frac{2\pi\Phi}{\Phi_0}\right)^2 + \varepsilon_s(p), \tag{14}$$

where p is the quasi momentum and s is the band index of the corresponding CPB Hamiltonian [4]. In the IST qubit case, where the E_J/E_C is in the transmon limit the $\varepsilon_s(p)$ can be written as [2]

$$\varepsilon_s(p) \simeq E_s(p = 1/4) - \frac{\epsilon_s}{2}\cos(2\pi p). \tag{15}$$

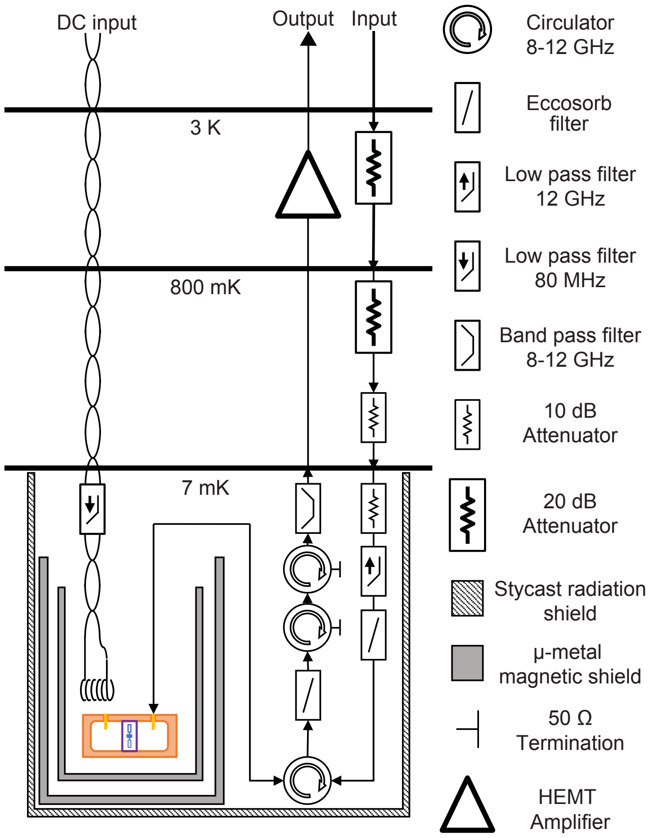

Supplementary Figure. 3. **Cryogenic measurement setup.** Shows the microwave components used in different stages of the dilution refrigerator used to control and readout of the IST qubit.

Deep in the transmon limit, ϵ_s is exponentially suppressed [2] and therefore the Hamiltonian in equation (14) represents the IST qubit as a free particle with a quadratic dispersion in external flux. In Supplementary Figure 4b, the lowest state of a corresponding well is identified by simply fitting a quadratic function. Therefore the functional dependence of the first transition was obtained by subtracting the two fitted parabola to the numerical data, which was then used to fit the experimental data presented in Fig. 5.

It is important to note that the correct qubit fit parameters for devices A and B were obtained by also including the coil parasitic modes which were found close to the first transition frequency. In case of device A, $\nu_{p01} = 6.1222\,\text{GHz}$ and the parasitic mode $\nu_p = 6.1890\,\text{GHz}$ with a coupling of 15.9 MHz, while for device B the first qubit transition was at 6.296 GHz and the parasitic mode located at 6.13 GHz with a coupling of 22 MHz. In case of device C the parasitic mode was at sufficiently high frequency to not affect the fitting procedure.

Another important factor for obtaining accurate numerical results for IST matrix elements, specially for flux transitions because of their extremely small values as low as 10^{-13} , is to use a sufficiently high cutoff value for the number of harmonic oscillator basis functions used to carry out the calculations. In all the simulations shown in the paper such as Fig. 1, 2 and 3 of the main text we used a cutoff value of 160 in the scQubits library to obtain accurate results.

SUPPLEMENTARY NOTE 5: QUANTUM JUMPS, QND-NESS AND FIDELITY

In this section we show measurements of quantum jumps and provide further details regarding our QND-ness and fidelity of the IST plasmons in high measurement power regime. The quantum trajectories allow instantaneous (limited by the measurement bandwidth and cavity line width) monitoring of the qubit state and therefore is another tool to study the QND-ness of the measurement. The pulse sequence for observing quantum jumps as well as the fidelity and QND-ness experiment are provided in Supplementary Figure 5a. A hundred of such quantum trajectories are shown in Supplementary Figure 5b, with a measurement power corresponding to 627 photons in the cavity and

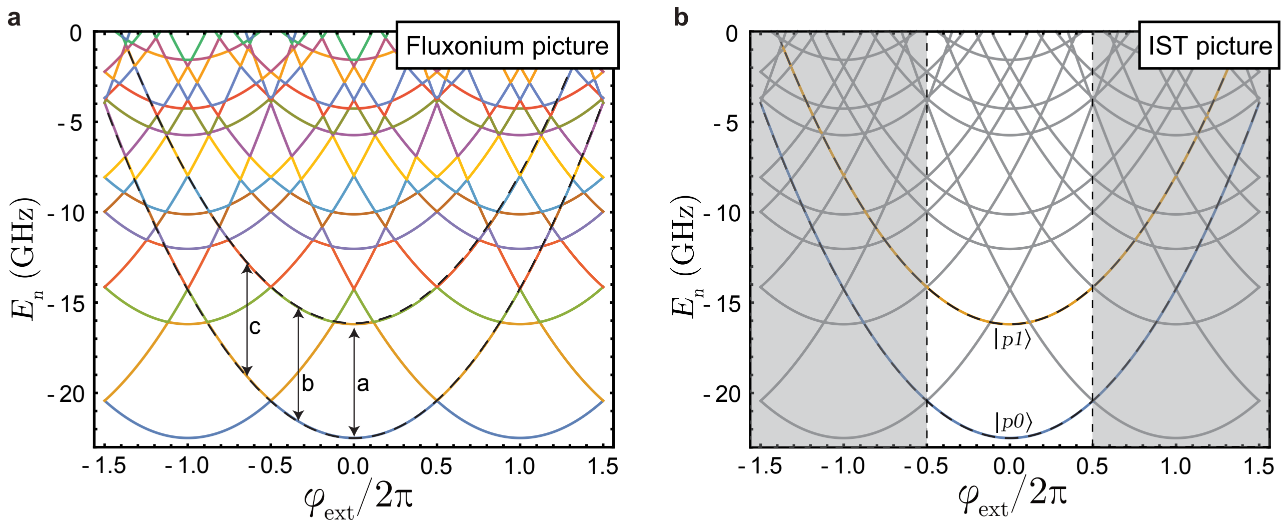

Supplementary Figure. 4. **Numerically calculated eigenenergies of device B. a**, Eigenenergies color-coded by their respective state number m. The black dashed line guides the eye on the two lowest states located in one specific potential well and is measured experimentally as the first transition. Position a labels the transition correctly as $|p0\rangle \rightarrow |p1\rangle$, while in position b and c the desired transition refers to $|p0\rangle \rightarrow |p2\rangle$ and $|p1\rangle \rightarrow |p3\rangle$ respectively. The reason for this is that in the fluxonium flux transitions are allowed and the system is always found in its global minimum while in the IST qubit the phase particle is trapped in a stable flux configuration and stays in one specific well. **b**, A quadratic fit to the ground to first excited state transition of the IST qubit (dashed lines). In the IST regime all the eigenenergies are represented by a parabola vs. flux, which makes the fitting process significantly easier.

2 MHz measurement bandwidth, for qubit prepared in ground and excited state respectively. The color map of each trace shows the amplitude of the quadrature rotated to reflect the excited state as high amplitude (yellow) and ground state as low amplitude (green). The non-QND effects are clear in 4 traces were the qubit was prepared in ground state. Also the decay of the qubit is apparent in traces were the qubit was prepared in the excited state.

Taking two data points (500 ns apart) as separate measurements of the qubit state, we can calculate the QND-ness once again and sweep the measurement power to extract the Supplementary Figure 5c. The maximum of the QND-ness is once again confirmed to be 92.5% as Fig. 7a however the position moved down to 560. We believe this is due to 500 ns readout pulse used in both QND-ness and fidelity experiment which does not allow the cavity to reach the steady state photon number. If we assume that the cavity reaches steady state at roughly $10/\kappa \approx 1.5$ μs, then a 500 ns pulse requires three times the power to reach the same photon number, hence explaining the occurrence of the maximum in QND-ness and fidelity in Fig. 7 at approximately 1500 photons. Increasing the measurement bandwidth to 4 MHz and reducing the readout pulse length to 250 ns pushes the maximum of the QND-ness to two times measurement power corresponding to approximately 3500 photons as expected.

Finally we show the IQ histograms for photon number 1437 and 2556 in Fig. 7 to give an overview of our measurement limitations. At photon number 1437, when the qubit is initialized at ground state, less than 1% of the population are scattered in plasmon excited state (possibly thermally excited) and $1-2\%$ are located in another state which we believe to be fluxon state. Applying the π-pulse brings 87% of the population to the excited state. The remaining 13% are due to the limited T1 of the qubit and slow readout (about $7-8\%$ decay to ground state after 500 ns) and the rest is possibly preparation error caused by the poor shape of the applied π-pulse. At photon number 2556 the emergence of the third state is clear which we believe to be the excited fluxon state.

SUPPLEMENTARY NOTE 6: EXCITED FLUXON STATE PREPARATION

In this section we study the IST qubit with the pump power at the three flux biases where we reported the long decay rates shown in Fig. 8c. Starting at the zero external flux (color green in Fig. 8c), a two tone spectroscopy experiment while sweeping the pump power reveals Supplementary Figure 6a. At low pump power the qubit starts at the well with lowest energy (detected with the plasmon frequency of 6.706 GHz) and as the pump power increases to the range of $32-161$ photons in the cavity, the measurement baseline dramatically changes and a new plasmon frequency of 6.56 GHz (matching the plasmon frequency of the first neighbouring well) is detected. This is followed by a reset of the fluxon to the ground state in the power range of $200-800$ photons (where we perform high QND

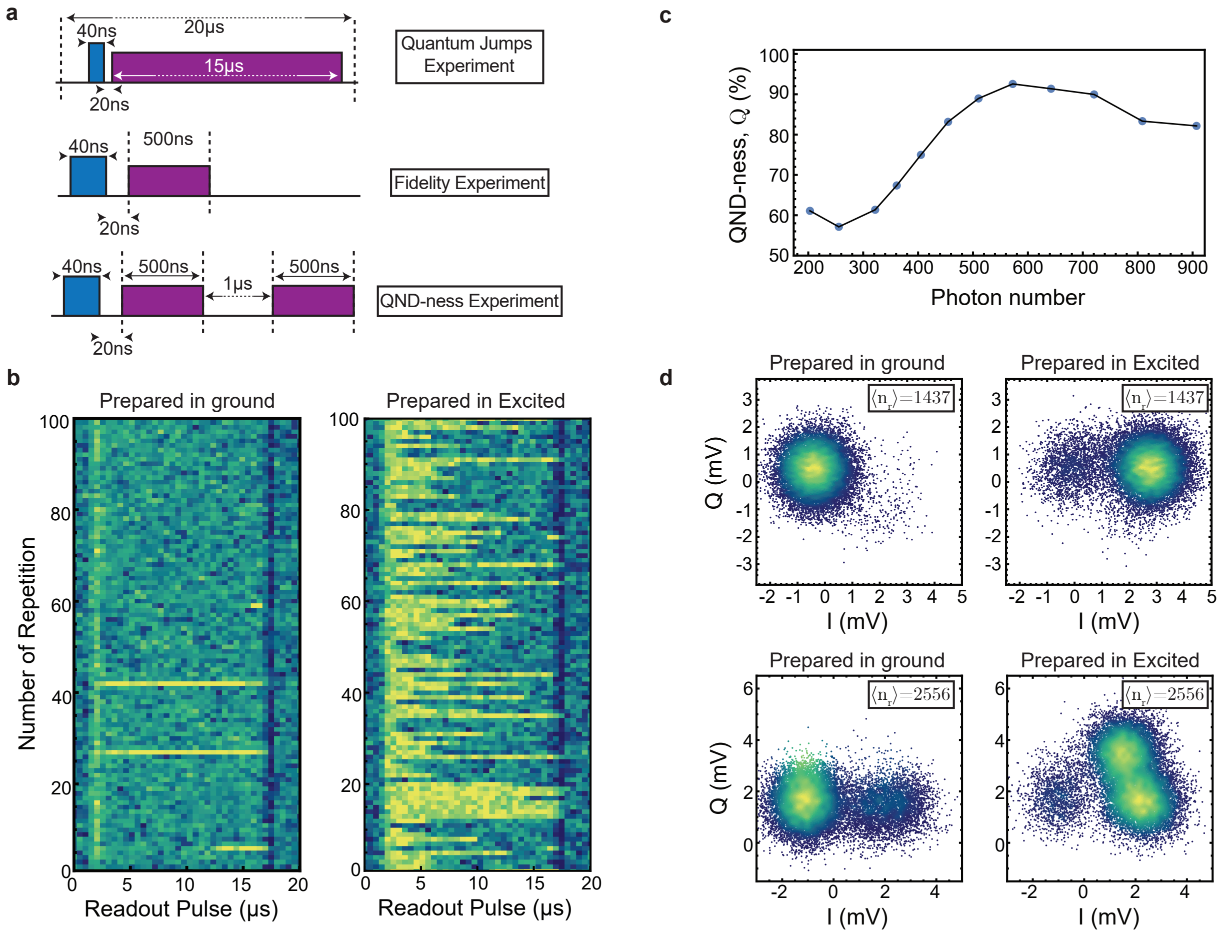

Supplementary Figure. 5. **Supplementary figure for high measurement power experiments.** **a**, The pulse sequence and length for observing quantum jumps, fidelity (Fig. 7a) and QND-ness (Fig. 7b) experiments. **b**, Hundred traces of a 15 μs long readout pulse showing quantum jumps with qubit prepared in ground and excited state respectively. These traces were measured using 627 photons in the cavity and a 2 MHz measurement bandwidth. **c**, The calculated QND-ness using quantum jumps in steady state, showing a more realistic photon number for performing a QND measurement. **d**, Showing the IQ histograms for photon number 1437 (high QND and fidelity) and 2556 (non-QND and low fidelity) respectively.

and fidelity of the plasmon state). At photon number above 1000, we observe both spectroscopy picks at 6.56 and 6.706 GHz together indicating a mixture of $|f_0\rangle$and $|f_1\rangle$ states. Averaging 200 traces with plasmon drive fixed at 6.56 GHz (probing the probability to find the system at $|f_1\rangle$) results in Supplementary Figure 6b. This figure identifies a pump pulse with power corresponding to 72 photons as the excitation pulse. Regardless of the initial state of the system, applying a pulse with a power in the range of $200-800$ resets the system to $|f_0\rangle$ state. Using the described calibrated pulses, we were able to switch between excited and ground fluxon state with an error rate lower than 3%. The Supplementary Figure 6c and d shows the same experiment performed for the flux biases color coded with yellow and purple in Fig. 8c respectively. As can be seen in Supplementary Figure 6d, near the half of flux quanta only a mixture of fluxon states can be prepared. Sweeping the excitation pulse length shows a smooth transfer of population from $|f_0\rangle$ at zero length to fully excited at $10/\kappa \approx 1.5$ μs (photon number reaching the steady state in the cavity) and further increase of the pulse length keeps the system at $|f_1\rangle$, therefore in all experiments a pump pulse of 10 μs is used.

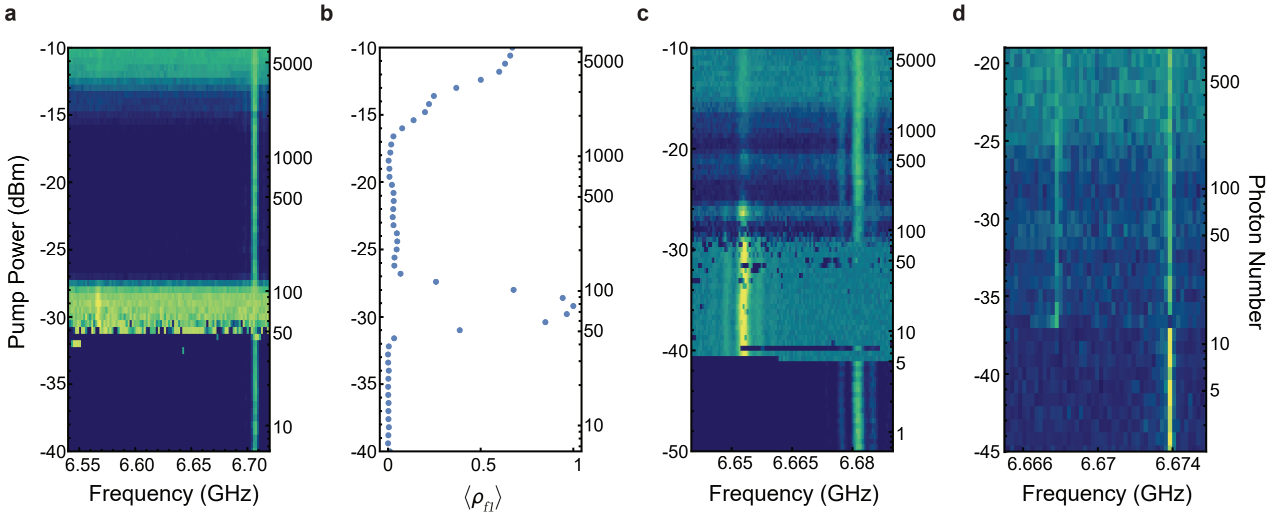

Supplementary Figure. 6. **Fluxon state preparation versus resonator pump power.** **a**, Shows an averaged two-tone spectroscopy measurement right after a 10 μs long resonator pump pulse vs. plasmon probe frequency and resonator pump power, performed at zero flux (color coded green in Fig. 8c). **b**, Averaged measurement of 200 traces at a fixed plasmon probe frequency of 6.56 GHz. This measurement clearly identifies the resonator pump power corresponding to 72 photons as the optimal excitation condition. The pump power corresponding to the range of 200 − 800 photons in the cavity can be used as a reset pulse. Also this range corresponds to the limit where high fidelity and QND readout of the plasmon states are possible. **c** and **d**, Show the same experiment as **a** performed for flux biases color coded yellow and purple respectively in Fig. 8c.

[1] D. J. Griffiths and D. F. Schroeter, *Introduction to quantum mechanics* (Cambridge University Press, 2018).

[2] J. Koch, T. M. Yu, J. Gambetta, A. A. Houck, D. I. Schuster, J. Majer, A. Blais, M. H. Devoret, S. M. Girvin, and R. J. Schoelkopf, Charge-insensitive qubit design derived from the Cooper pair box, Physical Review A **76**, 042319 (2007).

[3] G. J. Dolan, Offset masks for lift-off photoprocessing, Applied Physics Letters **31**, 337 (1977).

[4] J. Koch, V. Manucharyan, M. H. Devoret, and L. I. Glazman, Charging Effects in the Inductively Shunted Josephson Junction, Physical Review Letters **103**, 217004 (2009).
